# Preparation of Hydrophobic PET Track-Etched Membranes for Separation of Oil–Water Emulsion

**DOI:** 10.3390/membranes11080637

**Published:** 2021-08-17

**Authors:** Ilya V. Korolkov, Asiya R. Narmukhamedova, Galina B. Melnikova, Indira B. Muslimova, Arman B. Yeszhanov, Zh K. Zhatkanbayeva, Sergei A. Chizhik, Maxim V. Zdorovets

**Affiliations:** 1 L.N. Gumilyov Eurasian National University, Satpaev str. 5, Nur-Sultan 010008, Kazakhstan; i.korolkov@inp.kz (I.V.K.); narmukhamedova99@gmail.com (A.R.N.); bazarbaykyzy@list.ru (I.B.M.); arman_e7@mail.ru (A.B.Y.); zhanna01011973@mail.ru (Z.K.Z.); 2 The Institute of Nuclear Physics, Ibragimov str. 1, Almaty 050032, Kazakhstan; 3 A.V. Luikov Heat and Mass Transfer Institute of the National Academy of Sciences of Belarus, P. Brovki str., 15, 220072 Minsk, Belarus; Chizhik@gmail.ru; 4 Ural Federal University, Mira str. 19, 620002 Ekaterinburg, Russia

**Keywords:** hydrophobic modification, track-etched membranes, water–oil separation, silane

## Abstract

The paper describes the separation of an oil–water emulsion by filtration using poly(ethylene terephthalate) track-etched membranes (PET TeMs) with regular pore geometry and narrow pore size distribution. PET TeMs were modified with trichloro(octyl)silane to increase their hydrophobic properties. Conditions for the modification of PET TeMs with trichloro(octyl)silane were investigated. The results of changes in the pore diameters and the contact angle depend on the concentration of trichloro(octyl)silane and the soaking time are presented. The obtained samples were characterized by FTIR, AFM, SEM-EDX and gas-permeability test. Chloroform–water and cetane–water emulsions have been used as a test liquid for oil–water separation.

## 1. Introduction

Rapid growth in the oil and gas, petrochemical, metallurgical and food processing industries has resulted in a large production of oily wastewater. More than 75% of oil pollution gets into the hydrosphere during “normal” accident-free situations due to the imperfect technologies. The sources of pollution of water reservoirs with oil products are industrial effluents of enterprises of the petrochemical industry, metallurgy, mechanical engineering, as well as storm water, accumulating pollution from roads and territories of motor transport enterprises, gas stations, car washes and other services of the municipal services. The treatment of oily wastewater is an actual challenge [1]. Additionally, there is a problem of treatment of waste oils, solvents and other petroleum products for reuse [2]. The choice and effectiveness of oil/water separation methods depends not only on the size of the oil droplets, but also on other factors such as an oil concentration and chemical composition [3]. For these purposes, various methods can be used, such as flotation, coagulation, extraction, biodegradation and membrane technologies. According to a literature review [4], the overwhelming number of publications is devoted specifically to membrane separation methods.

Membrane technologies for the separation of oil–water emulsions have advantages, consisting in a higher permeate quality, the possibility of separating diluted stable emulsions, a smaller plant footprint, easier automation, no need for chemicals and, therefore, a decrease in waste and energy consumption [5]. These advantages, combined with the ability of the membranes to separate other contaminants present in solution, make the membranes competitive with other technologies. In the process of water–oil mixtures separation the following types of membrane technologies are used: membrane distillation, osmosis, nano-, micro- and ultra-filtration [6]. Various types of membranes are used: flat, hollow fiber and tubular, made of various polymers, ceramics, porous glass, etc. When choosing a membrane, the following characteristics should be taken into account: membrane porosity, membrane pore size distribution, and hydrophilicity/hydrophobicity of the material.

Most of the works are devoted to the study of oil separation in water, where hexane, petroleum ether, toluene, or chloroform are used as oil. For example, authors [7] prepared superhydrophobic/super-oleophilic polystyrene-Fe_3_O_4_ nanofiber membrane by electrospinning with CA of 162° for efficient hexane-water emulsion separation (flux is 5000 L/m^2^h). Lin and coauthors [8] prepared fluorinated SiO_2_-sprayed PVDF membrane with CA of 171.8° for water–petroleum ether emulsion separation with high flux of 2379 L/m^2^h and efficiency of 99.94%. Emulsions with higher viscosity oil are less studied, separation of such emulsions with membranes leads to lower fluxes, for instance, Zhang et al. [9] tested polyethylene (PP) membrane with grafted poly(2-dimethylaminoethyl methacrylate) in diesel/water separation with fluxes of 20–60 L/m^2^h. Flux for gasoline/water is around 500 L/m^2^h, olive oil/water is around 200 L/m^2^h and sesame oil/water is around 100 L/m^2^h for PS@Fe_3_O_4_ membranes [7] with separation efficiency of 96–92%.

Now, track-etched membranes (TeMs) have a record narrow pore size distribution, which can have a positive effect on the selective properties of separation, leading to its improvement. TeMs are made from thin polymer films irradiated with a heavy ion beam at a DC-60 heavy ion accelerator. Then, the irradiated polymer is etched by chemicals, as the result, membranes with cylindrical pores with the diameter range of 0.02–5.0 μm and pore number up to 10^10^ pores per cm^2^ can be obtained. A characteristic feature and advantage of TeMs is the regular geometry of pores with the ability to control their number per unit area and narrow pore size distribution. This, in turn, provides the improved selectivity and specific performance of the membranes [10].

TeMs made from various polymers are widely used, for instance, in precision ultrafiltration and microfiltration of liquids and gas cleaning; in the system of analytical substances control; food, pharmaceutical and chemical industries; microelectronics; and other areas of science and industry [11]. At the same time, there is only one work [12] devoted to primary study of the possibility of using polycarbonate TeMs for the separation of water–oil mixtures. The effective use of TeMs in the separation of oil–water emulsions requires a significant expansion of the range of their characteristics (pore structure, hydrophobicity/hydrophilicity, the creation of special chemical groups on the surface).

In order to modify the surface of materials, various methods are used, such as coating, deposition, ozonation [13], plasma [14], laser, radiation treatment and methods of graft polymerization [15,16]. For hydrophobization, polysiloxane and fluorine-containing compounds are often used [17]. The main difficulty of hydrophobization is the creation of a strong bond between the hydrophobic layer and the membrane, which can be stable over time.

In this article, at the first time we present the results of PET TeMs hydrophobization by simple method of soaking in trichloro(octyl)silane solution via polycondensation reaction on membrane surface. Obtained hydrophobic PET TeMs were tested in separation of chloroform–water and cetane–water emulsions.

## 2. Materials and Methods

### 2.1. Chemicals

Trichloro(octyl)silane (TCOS), isopropanol and chloroform were supplied by Sigma Aldrich. All other chemicals and solvents such as *o*-xylene, cetane, ethanol, acetic acid and sodium hydroxide had purity of analytical grade. In all experiments, deionized water (18.2 MΩ) obtained from Aquilon-D301 was used.

### 2.2. Obtaining and Modification of Track-Etched Membranes

**Figure 1:**
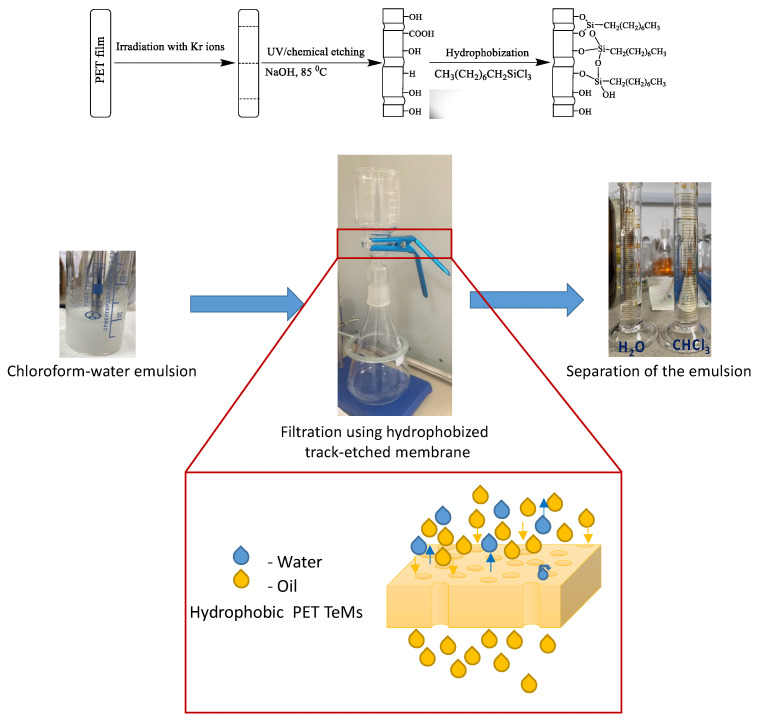
Scheme of PET track-etched membrane modification.

Poly(ethylene terephthalate) track-etched membranes (PET TeMs) were prepared by irradiation of PET film (12 µm thickness) by Kr ions on accelerator DC-60 (Nur-Sultan, Kazakhstan) with an energy of 1.75 MeV/nucleon and ion fluence of 1 × 10^8^ ion/cm^2^. Then, membranes were processed by photosensitization for 30 min from each side and chemically treated with 2.2 M NaOH at 85 °C for certain periods of time to prepare membranes with pore sizes of ~200, 250, 300 and 350 nm. Chemical treatment of PET film also led to hydrolysis of the ester groups of PET with breakage of the polymer backbone and formation of –COOH and –OH groups at the chain termini [18]. Modification of PET ion-track membranes was performed according to the scheme presented in Figure 1. For this purpose, PET TeMs with a different diameter were soaked in a solution of trichloro(octyl)silane in *o*-xylene in concentration range from 0.5 mM to 80 mM and time from 1 h to 24 h. Prepared membranes have thickness of 11.8 µm and porosity from 3 to 14%.

### 2.3. Methods of Characterization

FTIR spectrometer InfraLUM FT-08 was used to record FTIR spectra (range 400–4000 cm^−1^, 25 scans, 2 cm^−1^ resolutions on ATR accessory (PIKE, Madison WI, USA)). Pore size of the membranes was evaluated by gas flow rate measurement at a drop pressure of 20 kPa [19] using Equation (1):

(1) $$r^{3}=\frac{Q*3l}{\sqrt{\frac{2\pi }{RTM}}\Delta p4n}$$

where *r*—pore radius (m), Q—air capacity (m^3^/h), l—film thickness (m), ∆*p*—applied pressure (Pa), *R*—universal gas constant (J/mol*K), *M*—molar mass of air (kg/mol), *n*—surface pore density (irradiation fluence) (1/m^2^) and *T*—temperature (K).

Burst strength was evaluated at pressure that damages a circular sample of 1 cm^2^ surface area. For hydrophobized membranes with pore diameter of ~200 nm, burst strength is 0.420 MPa, for 250 nm-0.412 MPa, 300 nm-0.364 MPa and 350 nm-0.332 MPa.

EDX analysis was done using Hitachi TM 3030 with microanalysis system Bruker XFlash MIN SVE at 15 kV.

The CA was determined on a DSA 100E (KRUSS, Hamburg, Germany) by the sessile drop method based on the construction of a tangent at a three-phase point: liquid-substrate-air. Distilled water and diiodomethane (Sigma-Aldrich, 99% pure) were used as test liquids. The values of the free surface energy (γ) and its specific polar component (γ_p_) were calculated on the basis of the CA values using the OWRK method (Owens, Wendt, Rabel and Kaelble). The accuracy of the contact angle measurement is ±0.10.

The membrane surface structure and local mechanical properties (adhesion force and elasticity modulus) were studied on an atomic force microscope (NT-206, ALC «Microtestmachines», Gomel, Republic of Belarus) using standard silicon cantilevers NSC 11 A (Mikromacsh, Talinn, Estonia), stiffness 3 N/m and radius of curvature no more than 20.7 nm. The radius of curvature is measured using standard calibration sample TGT01 (Mikromacsh, Talinn, Estonia).

Based on the results of AFM scanning of 5 × 5 μm areas with a resolution of 256 × 256 points, *R_a_* and *R_q_* of the sample surface were determined in the SurfaceExplorer processing program. Average values were calculated over 10 scan areas. The *R_a_* index characterizes the variability in *Z* of the surface, within the selected area and it is calculated by Equation (2):

(2) $$R_{a}=\frac{1}{N}\overset{N_{y}-1}{\underset{j=0}{\sum }}\;\;\overset{N_{x}-1}{\underset{i=0}{\sum }}|Z_{i,j}-\bar{Z}|$$

where: *N*—number of scan matrix points; *Z_i,j_*—height in position (*x*, *y*); and $\bar{Z}$—arithmetic mean of the height in the entire scan matrix.

Root mean square deviation (*R_q_*) is calculated using Equation (3):

(3) $$R_{q}=\left(\frac{1}{N_{x}\cdot N_{y}}\overset{N_{y}-1}{\underset{j=0}{\sum }}\;\overset{N_{x}-1}{\underset{i=0}{\sum }}|Z_{i,j}-\bar{Z}|\right){}^{1/2}$$

The elasticity modulus (E) was calculated by AFM data on the base of curves of approach of the tip of the probe to the surface of the sample are given in the Static Force Spectroscopy mode using the Johnson–Kendall–Roberts model, which takes into account both of the adhesion forces and the elastic deformation of interacting objects model. The values of the adhesion force (Fa) were calculated based on the values of the detachment of the tip of the probe from the surface of the sample.

### 2.4. Oil–Water Separation

The filtration and separation ability of prepared PET TeMs for oil–water mixtures and pure solvents was elucidated by simple filtration set (Figure 1). A 6 cm diameter membrane was fitted into the filter, filtration area was 0.001256 m^2^. Filtration set was connected to vacuum pump with vacuum controller. Filtration was carried out at vacuum of 900, 800, 700 and 600 mbar. When the water-in-oil emulsion was filtered through the membrane, water was removed and the cleaned oil (chloroform or cetane) was collected in the beaker. Oil–water emulsion was prepared using Disperser IKA T 18 digital ULTRA-TURRAX (chloroform:water = 10:1 (vol.), cetane:water = 100:1(vol.)).

The flux (*F*) of the filtered oil was calculated using Equation (4):

(4) $$F=\frac{V}{S\cdot t}$$

where *V* is the volume of the oil that permeates through the membrane, *S* is the filtration area of PET TeMs, *t* is the time.

The volume of water collected after separation was measured and the separation efficiency (*R*, %) was calculated using Equation (5):

(5) $$R=\frac{V_{2}}{V_{1}}100\%$$

*V*_2_ is the volume of water collected after separation; *V*_1_ is the volume of water in water-in-oil emulsion before separation.

## 3. Results and Discussion

PET TeMs were hydrophobized by TCOS via the polycondensation reaction. It is based on the hydrolysis of Si-Cl bonds of silanes by OH groups of PET TeMs surface. Scheme of the reaction is presented in Figure 1. Optimization of concentration of TCOS in *o*-xylene and time of the reaction was carried out. The change in pore diameter (from gas flow method) and contact angle depending on the concentration of the agent is presented in Table 1.

As can be seen from Table 1, an increase in the contact angle is observed with an increase in the concentration of the TCOS to 15 mM, then, even with an increase in the concentration to 20 and 80 mM, the contact angle is decreased. This can be explained by the fact that molecules of TCOS at high concentrations react faster with each other than with the membrane surface, since the reaction is heterogeneous. Moreover, in FTIR spectra, Si-OH bonds were detected at 925 cm^−1^. The presence of such polar groups indicates the incompleteness of the reaction, which can reduce the contact angle [2]. Thus, at 15 mM of TCOS, the greatest hydrophobization is observed with preservation of the pore structure of the membrane.

The influence of reaction time on the contact angle is presented in Table 2. The reaction time has less influence on the contact angle than concentration of TCOS. The membranes have already become hydrophobic after 2 h of reaction, then, with an increase in the reaction time, a slight increase in the contact angle is observed up to 99°. The specific polar component of free surface energy and contact angle after 2 h of reaction have not been changed, but according to the FTIR spectroscopy data, the Si-OH groups are established on the modified membrane surfaces, which indicates the incompleteness of the reaction. So, optimal time of reaction is 24 h.

**Figure 2:**
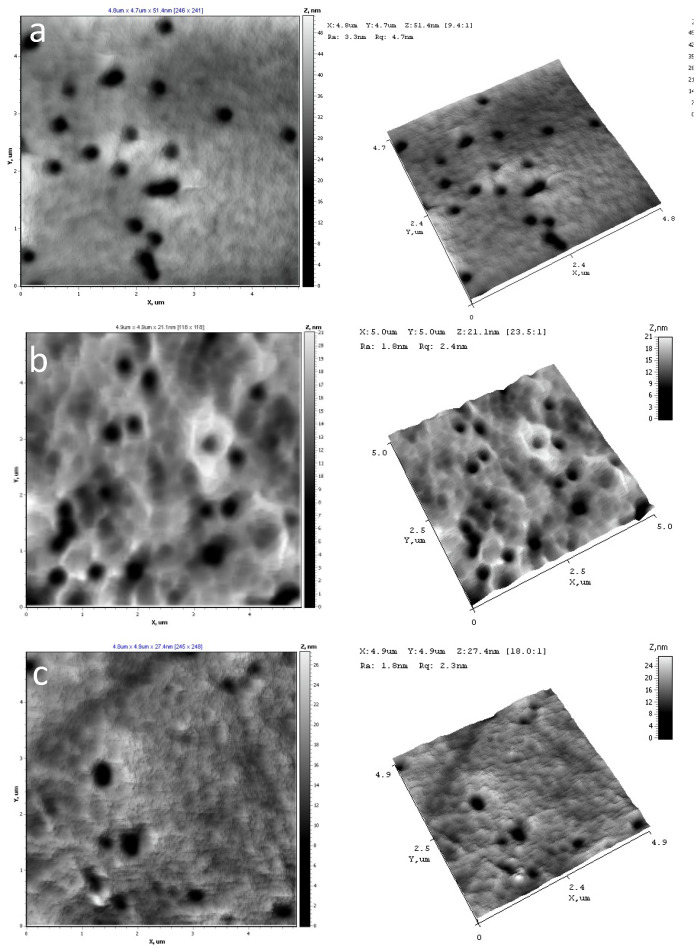

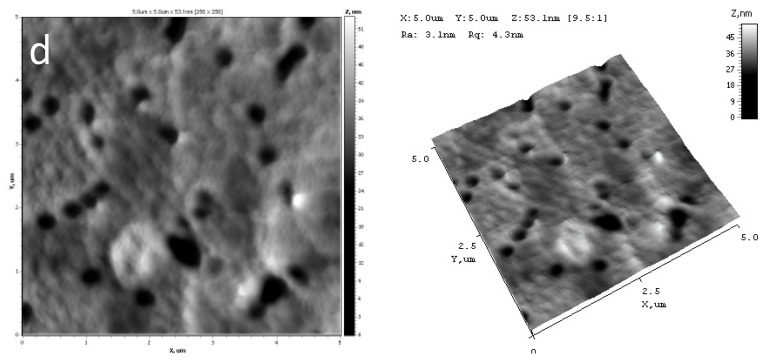
AFM of initial PET TeMs (**a**) and after modification at 15 mM TCOS for 2 h (**b**), 4 h (**c**) and 24 h (**d**).

AFM was used to visualize the surface of PET TeMs before and after modification as well as to calculate Ra, Rq indexes and adhesion strength. Results are presented in Figure 2 and Table 3.

It was shown, that after modification values of roughness (R_a_, R_q_) were increased slightly and not changed at different time of reactions. Elasticity modulus and adhesion forces values characterize mechanical properties of material surfaces in local point. In the case of modified membranes, the influence of PET TeMs substrates is significantly, as results the data are unchanged within experimental error. It can be assumed, that modifier and substrates were formed united materials at the expense of chemical bonds that is confirmed by FTIR-data.

According to AFM, pores of the initial and modified membranes are uncovered in investigated areas. After modification discrete 200 nm sized conglomerates can be observed at the surface. Structure variation is not observed at all the AFM scans.

**Figure 3:**
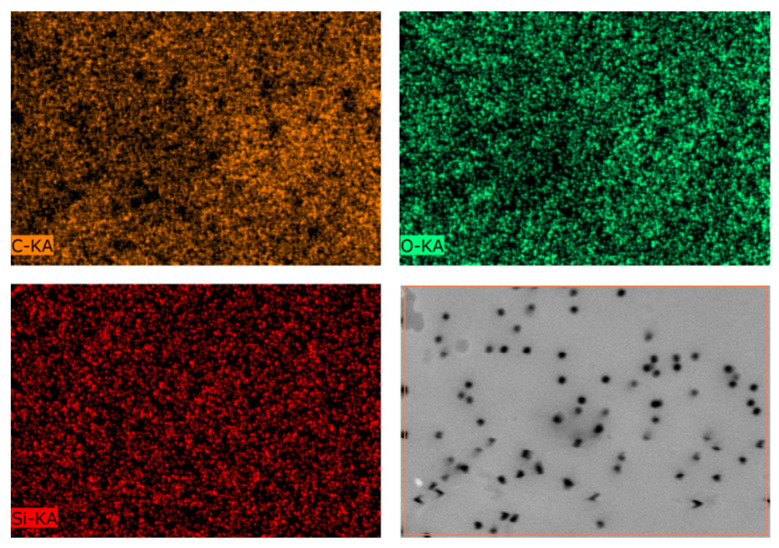
EDX mapping of elements and SEM picture of the surface of PET TeMs-TCOS modified at 15 mM and 24 h.

EDX mapping presented in Figure 3 shows us uniform distribution of elements on the membrane surface. Data collected from the EDX analysis are summarized in Table 4. It is seen that with increasing in the time of the reaction, content of Si and C is also increased from 1.5% and 75% to 5.6% and 82.9%, respectively.

**Figure 4:**
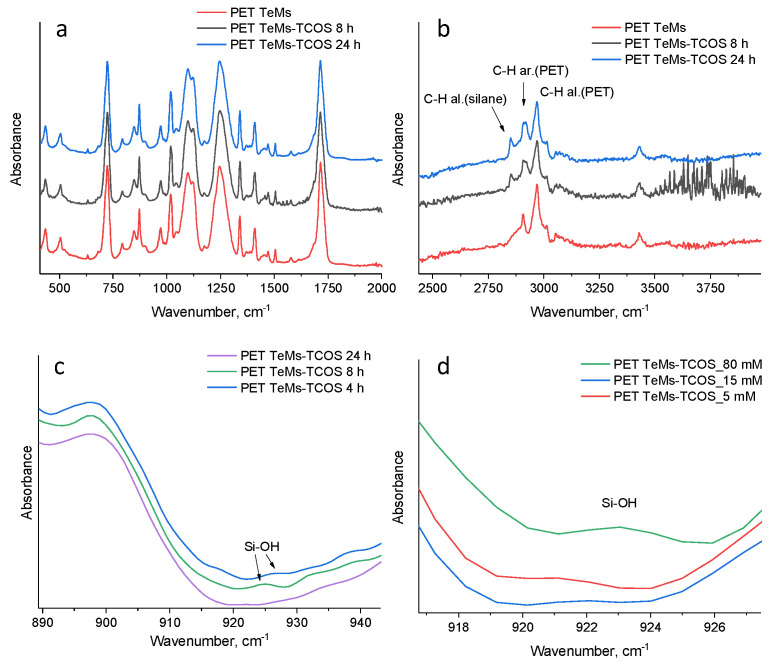
FTIR spectra of the initial and modified PET TeMs depends on time in the range of 400–2000 cm^−1^ (**a**), 2400–4000 cm^−1^ (**b**) and 890–940 cm^−1^ (**c**) depending on concentration (**d**).

FTIR spectroscopy have shown chemical changes on the membrane surface. Results are presented in Figure 4. The initial PET TeMs have absorption bands at 2970 cm^−1^ (benzene ring, CH), 2912 cm^−1^ (aliphatic CH), 1713 cm^−1^ (C = O group), 1615, 1470, 1430, 1409 cm^−1^ (aromatic vibrations of the carbon skeleton), 1340 cm^−1^ (O-CH), 1238 cm^−1^ (vibrations of bonds of ether groups C(O)-O) and 970 cm^−1^ (O-CH_2_). The modification of PET TeMs with TCOS led to the appearance of a peak at 2850 cm^–1^, which is related to stretching vibrations of aliphatic C–H bonds. For a quantitative assessment, the values of the I_2850_/I_1410_ band ratio indexes were calculated on the basis of respective peak intensity (I). Band ratio index I_2850_/I_1410_ for the PET TeMs-TCOS- 4 h is 0.054, PET TeMs-TCOS- 8 h—0.067 and PET TeMs-TCOS- 24 h—0.100. Thus, the maximum amount of silane is observed at 24 h of the reaction. Moreover, as it is seen from Figure 4b, at 4 and 8 h, peaks are observed at 925 cm^−1^ that is related to Si-OH. The absence of peak at 925 cm^−1^ after 24 h of the reaction allows us to conclude that the reaction of polycondensation completed [20]. In addition, it should be noted that the presence of Si-OH groups can lead to a decrease in the contact angle as it is seen in Table 2.

**Figure 5:**
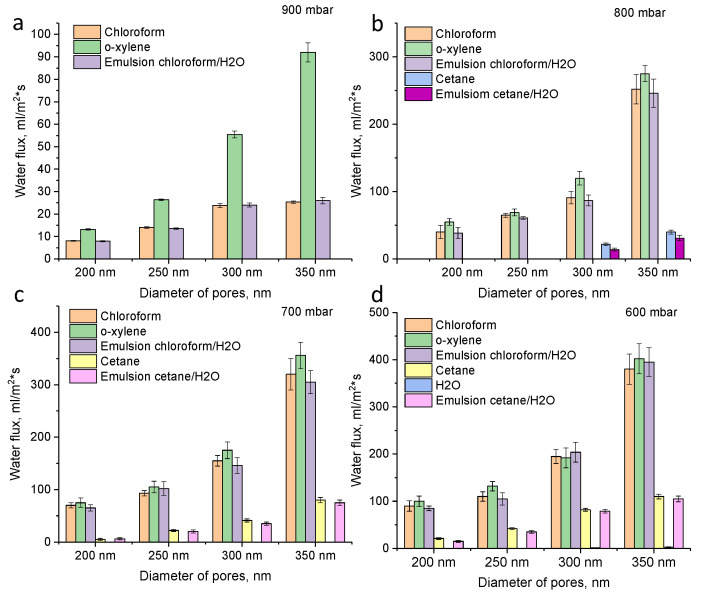
Fluxes of hydrophobic PET TeMs for chloroform, o-xylene, cetane and chloroform/water emulsion at different pore sizes (200, 250, 300 and 350 nm) and pressure 900 mbar (**a**), 800 mbar (**b**), 700 mbar (**c**) and 600 mbar (**d**).

Fluxes for pure liquids (o-xylene, chloroform, cetane, water) as well as oil–water emulsion (chloroform–water and cetane–water) for prepared membranes with different pore sizes (200, 250, 300 and 350 nm) and different pressures (900–600 mbar) are presented in Figure 5. It is seen that with an increase in the pore diameter of the membranes and a decrease in pressure, an increase in fluxes occurs. When the pressure drops from 900 to 800 mbar, there was a sharp increase in fluxes for membranes with a pore diameter of 350 nm from 35 mL/m^2^·s to 252 mL/m^2^·s (chloroform). Further, with decreasing pressure, there was a smoother increase in productivity, reaching the maximum value of 380 mL/m^2^·s at 600 mbar. However, at 600 mbar, it is seen that water can also penetrate through membranes (for membranes with 350 nm, water flux is 2.5 mL/m^2^·s). Thus, at 600 mbar, the degree of water–oil purification is low. The optimum vacuum pressure is 700–800 mbar.

**Figure 6:**
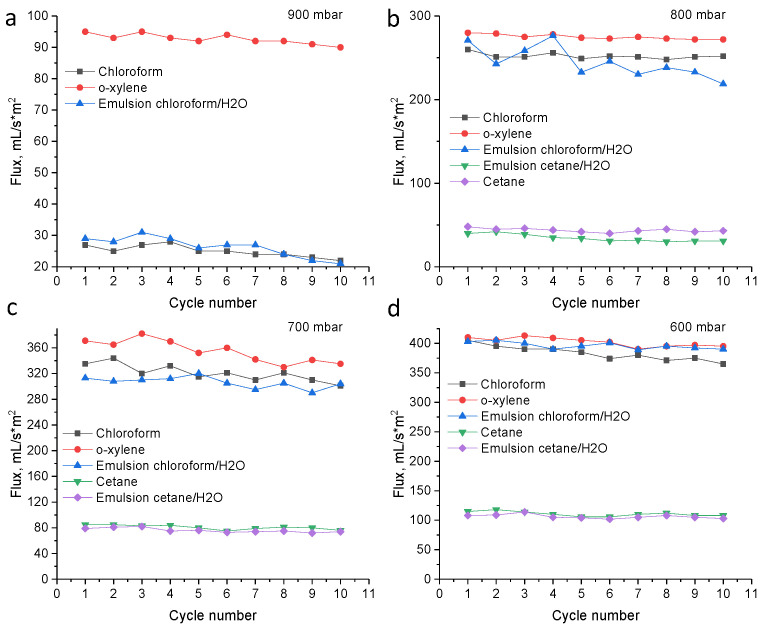
The fluxes of hydrophobic PET TeMs for various solvents and oil/water mixtures after each cycle (pore diameter of 350 nm) at pressure 900 mbar (**a**), 800 mbar (**b**), 700 mbar (**c**) and 600 mbar (**d**).

Oil–water separation was performed on the example of chloroform–water and cetane–water emulsions prepared using disperser. Since there is an excess water, after 5 min a small amount of water floated to the surface (in case of chloroform-water emulsion), however, complete separation of the emulsion occurred only after several hours. The use of a hydrophobic PET TeMs can separate the emulsion in a few minutes with high separation efficiency (99.9–99.5%). The stability of the hydrophobic membrane was studied for 10 cycles, results are presented in Figure 6. Only a slight decrease in fluxes is observed, in general the membranes remain stable, the degree of separation does not change. Comparing the flux of pure solvents and emulsions, the fluxes of pure solvents is slightly higher than that of emulsions. The fluxes of *o*-xylene and chloroform is higher than that of cetane, since cetane has a higher molecular weight and viscosity. In addition, it should be noted, that initial PET TeMs were also tested in emulsion filtration; however, separation efficiency was close to zero. It was observed that, first, chloroform passes through the pores of the membrane, then the water is also filtered out.

Obtained results in terms of flux and separation efficiency were compared with the results obtained by other research groups. Results are presented in Table 5. Prepared membranes have potential to be used for oil/water separation. Developed membranes have a high flux for water/chloroform emulsion and average flux for water/cetane with high degree of separation efficiency.

## 4. Conclusions

In this study, we have shown simple and technologically convenient method of PET TeMs hydrophobization with trichloro(octyl)silane. Hydrophobization at optimal parameters leads to CA up to 99°. Gas permeability and SEM have shown us only slight change in pore sizes, EDX analysis have confirmed uniform distribution of the agent over the surface. Hydrophobized PET TeMs was tested in oil–water separation. Membranes have shown stable fluxes and separation degree during 10 filtration cycles for chloroform-water and cetane-water emulsions. There was a dependence between flux and pore size and vacuum pressure. Optimal pressure was 700 mbar, at this pressure, maximum flux has been achieved with high separation efficiency. Membranes with a pore diameter of ~350 nm and vacuum pressure of 700 mbar have shown flux of 305 mL/m^2^h for chloroform–water and 75 mL/m^2^h for cetane–water emulsions. The results have shown showed a high potential for the possibility of using obtained membranes in oil–water separation.

## Figures and Tables

**Table 1:** Change in pore diameters and contact angle of the membrane dependence on the concentration of TCOS (time is 24 h).

Concentration of TCOS, mM	Effective Pore Diameter, nm	CA,°
Initial PET TeMs	240 ± 2	74.9
0.5	241 ± 3	83.0
1	240 ± 4	87.5
5	234 ± 5	87.2
10	239 ± 5	91.0
15	224 ± 5	99.0
20	215 ± 5	94.4
80	211 ± 9	86.0

**Table 2:** Change in effective pore diameter and contact angle of membrane depending on the time of reaction with TCOS (at a constant concentration of 15 mM).

Time, h	θ, Water	θ, Diiodomethane	γ, mJ/m^2^	γ_p_, mJ/m^2^	Effective Pore Diameter, nm
0	74.9	24.6	50.2	3.9	240
1	86.4	58.0	33.2	3.4	232
2	94.0	29.6	44.5	0.2	234
4	97.9	24.5	46.4	0.1	238
8	96.9	39.7	40.1	0.3	230
24	99.0	33.7	42.7	0.1	224

**Table 3:** Results of atomic force microscopy.

Sample of PET TeMs (250 nm) Modified at Different Time	R_a_, nm	R_q_, nm	E, MPa	F_a_, nN
Initial PET TeMs	4.5±1.7	8.0 ± 2.2	140.5 ± 16.5	42.3 ± 4.0
1 h	5.9 ± 1.2	7.7 ± 1.6	138.8 ± 18.0	38.7 ± 4.8
2 h	6.5 ± 1.6	8.5 ± 1.9	136.7 ± 18.1	42.8 ± 5.9
4 h	4.3 ± 1.7	5.8 ± 1.5	113.4 ± 20.4	57.2 ± 3.8
8 h	4.9 ± 1.4	6.1 ± 1.5	132.6 ± 23.6	51.0 ± 5.8
24 h	5.8 ± 1.1	7.4 ± 1.4	142.9 ± 22.57	44.9 ± 3.3

**Table 4:** Atomic content calculated based on EDX spectra.

Sample	Atomic Content, %		
	C	O	Si
Initial PET TeMs	72	28	-
PET TeMs-TCOS, 1 h	75	23.5	1.5
PET TeMs-TCOS, 8 h	79	18.8	2.2
PET TeMs-TCOS, 24 h	82.9	11.5	5.6

**Table 5:** Comparison of experimental results with other described in the literature.

Membrane	Emulsion Content	Flux, L/m^2^h	Separation Efficiency, %	Reference
Polyethylene (PP) membrane grafted with poly(2-dimethylaminoethyl methacrylate)	Diesel-in-water	60–20	80–100	[9]
Cellulose acetate/Nylon 66/Dimethyl Sulfoxide (D1)	Hexane–water	33	89	[21]
Cellulose acetate/Nylon 66/Formic Acid	Hexane–water	23	95	[21]
Polystyrene@ Fe_3_O_4_ nanofiber membrane	Hexane–water	5000	96	[7]
Polystyrene@ Fe_3_O_4_ nanofiber membrane	Gasoline	500	96	[7]
Polystyrene@ Fe_3_O_4_ nanofiber membrane	Olive oil	200	94	[7]
Polystyrene@ Fe_3_O_4_ nanofiber membrane	Sesame oil	100	92	[7]
Fluorinated SiO_2_-sprayed PVDF membrane	Water–petroleum ether	2379	99.94	[22]
Polypyrrole-coated mesh	Water/dichloroethane	500	99.9	[8]
ZnO-Co_3_O_4_ overlapped membrane	Water/toluene	67	99.97	[23]
PET track-etched membrane	Water/chloroform	1098 (pressure 700 mbar)	99.9–99.5	This work
PET track-etched membrane	Water/Cetane	270 (pressure 700 mbar)	99.9–99.5	This work

## Data Availability

The data presented in this study are available on request from the corresponding author.
